# Thyroid Eye Disease, Glycemic Control, and the Role of a One-Stop Clinic Approach: A Retrospective Audit Study

**DOI:** 10.7759/cureus.107831

**Published:** 2026-04-27

**Authors:** Keiko Carter, Zahid Khan, Bashir Mahamud, Furhana Hussein, Adnan Abdullah, Godwin Simon, Gideon Mlawa

**Affiliations:** 1 Internal Medicine, University Hospital of Wales, Cardiff, GBR; 2 William Harvey Institute, Queen Mary University of London, London, GBR; 3 Cardiology, University of South Wales, Pontypridd, GBR; 4 Cardiology, University of Buckingham, London, GBR; 5 Cardiology, Barts Heart Centre UK, London, GBR; 6 Internal Medicine and Diabetes and Endocrinology, Barking, Havering and Redbridge University Hospitals NHS Trust, London, GBR; 7 Internal Medicine, Barking, Havering and Redbridge University Hospitals NHS Trust, London, GBR

**Keywords:** british oculoplastic surgery society, dysthyroid optic neuropathy, electronic patient-reported outcome, european group on graves’ orbitopathy (eugogo), graves´disease, hyperthyroidism, retrospective observational study, steroid-induced hyperglycaemia, thyroid eye disease amsterdam declaration implementation group uk, thyroid eye disease (ted)

## Abstract

Background: Thyroid eye disease (TED) is a serious medical condition observed in patients with thyroid disorders, and patients often present in emergencies, threatening their vision.

Methodology: This retrospective audit was conducted at Queens Hospital, Romford, and included patients with TED who attended a dedicated ophthalmology-led TED clinic between January 2018 and June 2024. A total of 221 patients were included in this study, and their data were extracted from electronic and paper medical records, including clinic letters. Baseline and post-treatment glycemic monitoring was incompletely documented among steroid-treated patients (n=29). Baseline fasting glucose levels prior to steroid initiation were documented in 12 of 29 (41.4%) patients, and follow-up fasting glucose levels after steroid completion were documented in five of 29 (17.2%) patients.

Results: This study included 221 patients who visited the ophthalmology-led TED clinic between January 2018 and June 2024. The study cohort comprised 69% female and 31% male patients, with a mean age of 56.52 years (range, 22-92 years). Of the overall cohort, 29 patients (13.1%), aged 29-85 years, who received oral or intravenous systemic glucocorticoids for TED, were included in the main analysis group. Within this subgroup, four of 29 patients (13.8%) had pre-existing diabetes.

Conclusion: Glucose and HbA1c monitoring during systemic glucocorticoid therapy in patients with TED at this center was incomplete and fell below evidence-based recommendations, despite the recognised risk of steroid-induced hyperglycemia and new-onset diabetes with high-dose steroid exposure. There is a need for more standardised and improved care for patients with TED presenting to outpatient clinics. We recommend setting up a “one-stop” TED clinic to provide standardised care.

## Introduction

Thyroid eye disease (TED) is widely recognised as the predominant extrathyroidal feature of Graves’ disease [1-4]. Patients with TED can experience symptoms ranging from pain and double vision to visible changes in appearance, and in severe cases, vision loss due to dysthyroid optic neuropathy (DON) [5-8]. This condition is associated with increased morbidity, as large-scale studies have identified notable rates of strabismus and the need for surgical intervention [9,10]. In addition to physical symptoms, Graves’ disease with orbitopathy can lead to significant psychological effects; data from national registries show a higher risk of suicide in patients with both Graves’ disease and orbitopathy than in those without orbitopathy [9]. Although TED is most often linked to hyperthyroidism in Graves’ disease, it can also arise in euthyroid or hypothyroid autoimmune thyroid disease; however, the overall incidence in the general population remains low [8,10].

For individuals with active moderate-to-severe TED, expert guidelines suggest intravenous glucocorticoids or high-dose oral treatments as the primary therapy [5]. However, systemic glucocorticoids are known to cause high blood sugar levels, which can sometimes lead to new cases of diabetes and require careful risk assessment and ongoing monitoring [1,3,4,11]. Therefore, clinical guidelines advise checking baseline blood glucose levels, including glycated haemoglobin (HbA1c) when relevant, and recommend ongoing glucose monitoring and treatment plans tailored to the specific steroid regimen [3,4,12,13]. This is especially relevant for TED care involving high-dose intravenous methylprednisolone [5], as blood glucose levels may influence disease progression. Studies have also found that HbA1c is an independent risk factor for DON [12].

Although glucocorticoid treatment, blood glucose risk, and important TED outcomes are clearly linked, there is little published evidence on how consistently blood glucose is monitored in patients with TED receiving steroids, especially in clinics led by ophthalmologists [2,11]. UK recommendations highlight the need for multidisciplinary approaches to TED care [2], and practical studies support the use of structured services [11]. Despite this, the extent to which blood sugar risk assessment and monitoring are embedded in TED care routines seems to be inconsistent across real-world settings.

In this retrospective audit, we analysed data from an ophthalmology-led TED clinic focusing on the measurement and monitoring of baseline blood sugar levels, including the incidence of diabetes mellitus and HbA1c levels when available, among patients treated with systemic glucocorticoids for TED. Furthermore, we aimed to compare the current local practice of monitoring blood sugar and HbA1c levels in patients who had commenced glucocorticoid therapy against national and international guidelines [3,4,13,12]. A key aim of this audit was to highlight gaps in patient care and implement changes to improve patient care by aligning the current pathway with national guidelines for patients on steroids for TED [2,11].

## Materials and methods

Study design and setting

This retrospective audit was conducted at Queen's Hospital, Romford, United Kingdom (UK). The study focused on patients with TED requiring steroid therapy and patients with suspected or clinician-diagnosed TED who attended a dedicated ophthalmology-led TED clinic between January 2018 and June 2024 were included in the study. Ethics approval was granted by the local research and audit department of the hospital under the approval number (LN-097-24).

Inclusion criteria

The study included patients who were adults aged > 18 years, confirmed to have a diagnosis of TED and attended an ophthalmology-led TED clinic during the study period. 

Exclusion criteria

Patients who were younger than 18 years and patients attending the eye clinic for reasons other than TED were excluded from this audit. Furthermore, duplicate records and patients diagnosed with non-TED ocular disorders were also excluded from this audit. Finally, patients with missing data were also excluded from this audit. Before excluding the last group of patients, a thorough review of both electronic and paper notes was made to retrieve the missing data. 

Steroid-treated subgroup

A steroid-treated subject was defined as a patient who received systemic glucocorticoids (oral or intravenous) for TED during the study period, in accordance with the guideline-supported management of active moderate-to-severe disease [5]. Analyses of glucose and HbA1c monitoring focused on this subgroup because of the established risk of steroid-induced hyperglycemia and new-onset diabetes associated with systemic glucocorticoid exposure [1,3,4,13].

Data sources and data collection

Data were collected primarily from electronic patient-reported outcomes (EPRO) generated in the ophthalmology-led TED clinic. Additional information was obtained from correspondence with the endocrinology clinic and the Trust’s electronic laboratory reporting system, where relevant. Paper notes were reviewed if the electronic records lacked the required information. Four investigators performed data extraction and screening using a pre-defined data collection sheet. Any data discrepancy was resolved through mutual consensus and with input from the lead investigator. 

Variables and timing definitions

The collected variables included age, sex, baseline diabetes status (type specified where documented), and laboratory measures such as fasting blood glucose and HbA1c levels. For steroid-treated patients, the index date was defined as the date of initiation of systemic glucocorticoid therapy for TED. Baseline glucose and HbA1c levels were defined as the closest available laboratory values before steroid initiation. If multiple values were available, the result closest to the start of steroid therapy was selected.

Follow-up HbA1c

There was no protocolized time point for follow-up blood glucose testing in routine practice. Therefore, follow-up measures were defined as the first available laboratory results after the completion of systemic steroid therapy, irrespective of timing. The interval between steroid completion and follow-up testing (days) was recorded and summarised to describe variations in follow-up timing.

Standards for interpretation

The standard practice against which this retrospective audit was performed included "the European Group on Graves’ Orbitopathy (EUGOGO) guidance on systemic glucocorticoid therapy and safety considerations in TED" [5], and UK/international guidance on glucocorticoid-induced hyperglycemia recommends baseline glycemic assessment (including HbA1c, where appropriate) and ongoing glucose monitoring in patients exposed to high-dose systemic glucocorticoids [1,3,4,13].

Statistical analysis

Descriptive analyses were performed using Microsoft Excel (Redmond, WA, USA). Continuous variables are summarised as mean (standard deviation) or median (interquartile range), as appropriate. Categorical variables were summarised as counts and percentages. For the steroid-treated subgroup, the completeness of baseline and follow-up glucose and HbA1c testing was reported as n/N (%), stratified by baseline diabetes status, where feasible. The distribution of time from steroid completion to follow-up testing was summarised to reflect the absence of a fixed follow-up schedule.

## Results

**Figure 1:**
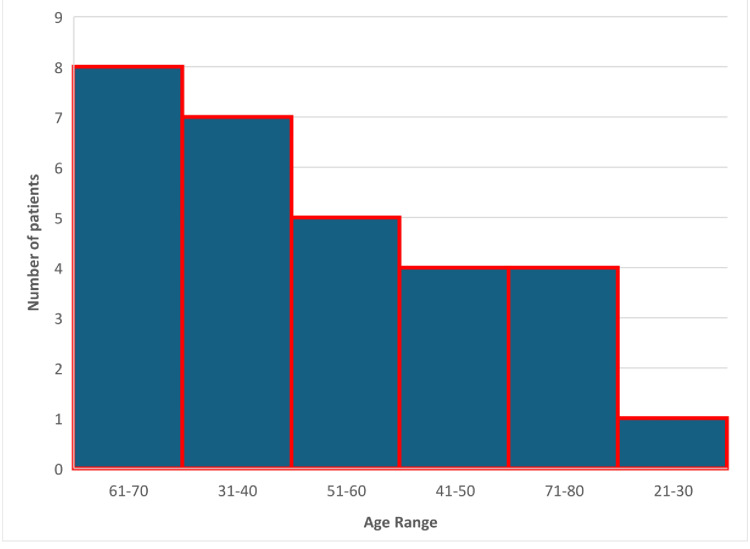
Histogram showing the age distribution of patients who received steroid therapy.

A total of 221 unique patients with TED, diagnosed or suspected, were seen at the ophthalmology-led TED clinic between January 2018 and June 2024. Data was retrieved for all the patients who attended the TED clinic. From the group of patients attending the TED clinic, 69% were female, and 31% were male, with a mean age of 56.52 years (range, 22-92 years). From this cohort of patients, only 29 patients (13.1%), aged 29-85 years, received oral or intravenous systemic glucocorticoids for TED, forming the main analysis group (Figure 1). The study participants' demographic data is presented in Table 1. 

**Table 1:** Study participants' demographic data.

Variable		Value
Total number of patients		221
Patients receiving steroids		29
Mean age ± standard deviation		56.52 ± 16.93
Gender	Female	153 (69%)
	Male	68 (31%)
Diabetes mellitus among the steroid-receiving group	Yes	4/29
	No	25/29
Hypertension	Yes	22/29 (75.8%)
	No	07/29 (24.2%)
Graves' disease	Present	24/29 (82.7%)
	Absent	05/29 (17.3%)
Baseline fasting glucose recorded	Yes	12/29 (41.4%)
	No	17/29 (58.62%)
Follow-up fasting glucose recorded	Yes	5/29 (17.2%
	No	24/29 (82.8%)
Baseline HbA1c recorded	Yes	20/29 (68.9%)
	No	09/29 (31.1%)
Follow-up HbA1c recorded	Yes	18/29 (62.1%)
	No	11/29 (37.9%)

**Figure 2:**
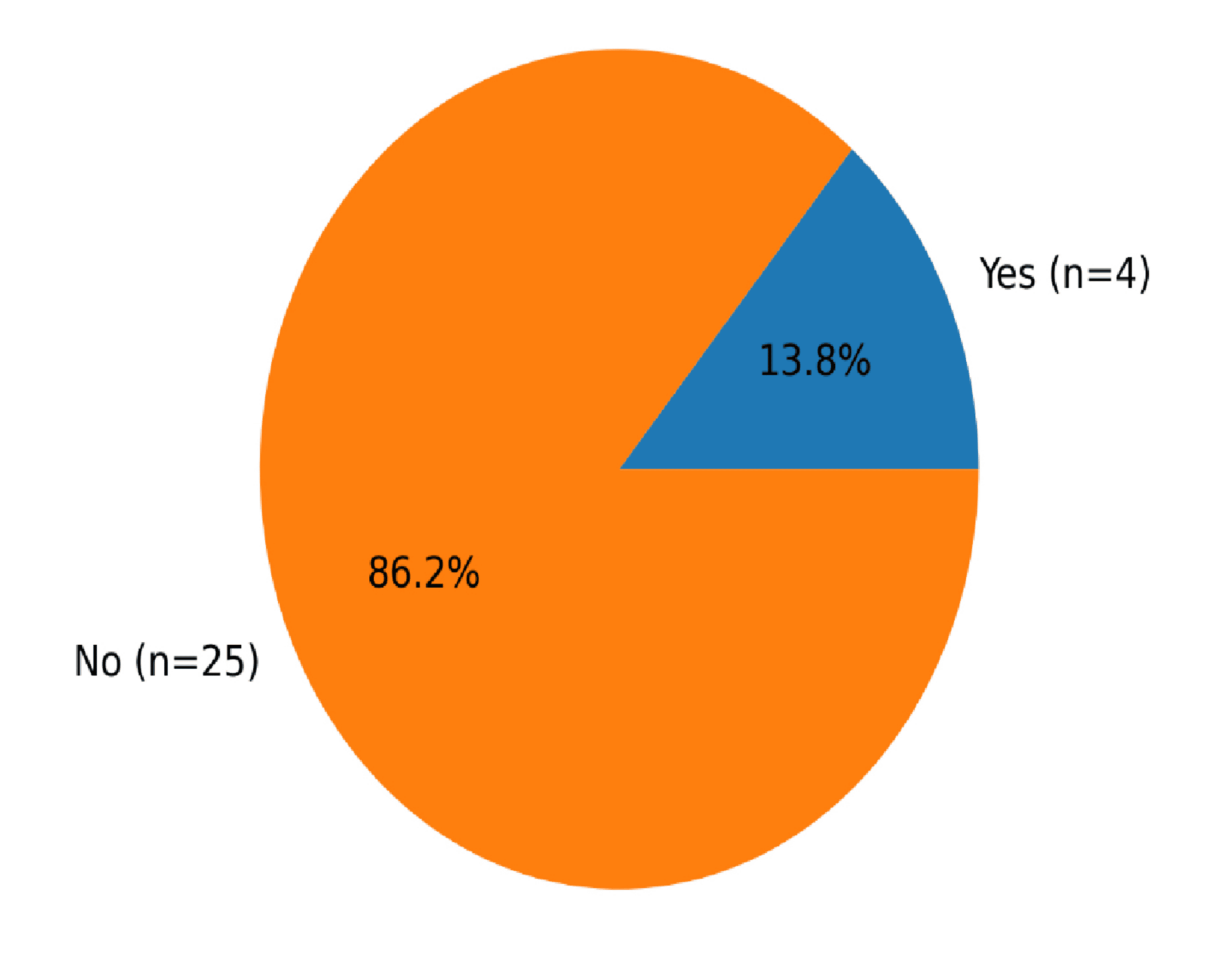
Pie chart showing pre-existing diabetes in steroid-treated thyroid eye disease patients.

**Figure 3:**
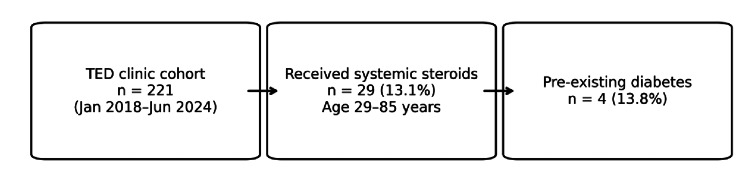
Overview of the cohort derivation and subgroup numbers. TED: thyroid eye disease

Within this subgroup, four of 29 patients (13.8%) had pre-existing diabetes, as shown in Figure 2. Figure 3 shows the study cohort flow, from the overall TED clinic population (n=221) to the steroid-treated subgroup (n=29; 13.1%; age 29-85 years), and the number of steroid-treated patients with pre-existing diabetes (n=4; 13.8%).

**Figure 4:**
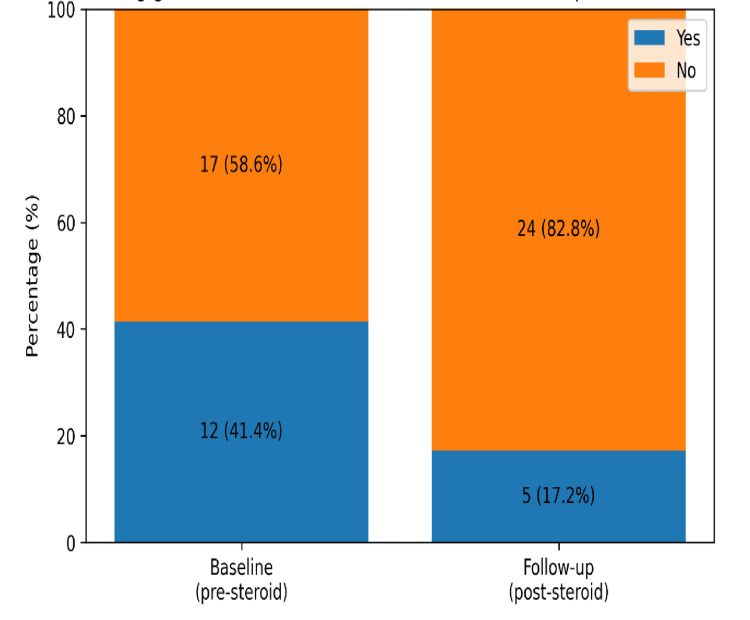
Bar chart showing baseline and follow-up fasting glucose documentation in steroid-treated thyroid eye disease patients.

**Figure 5:**
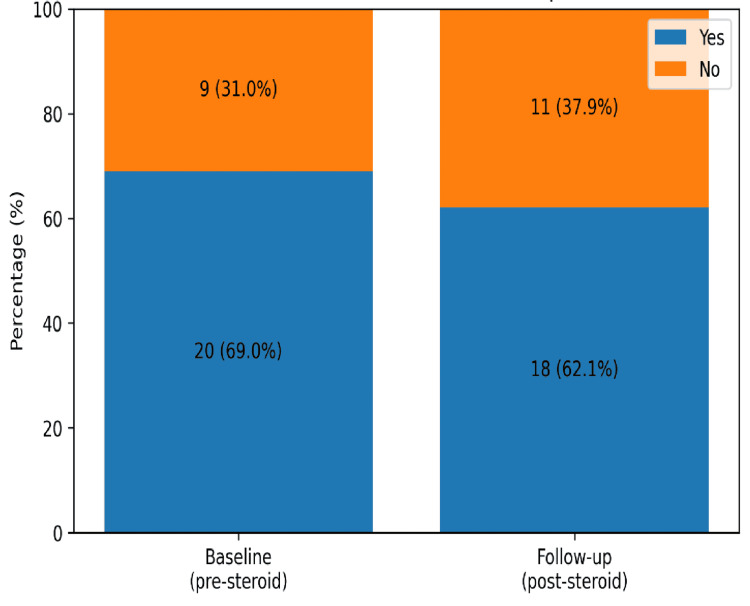
Bar chart showing documentation of baseline and follow-up HbA1c in steroid-treated thyroid eye disease patients.

There was a lack of documentation for a significant number of patients for baseline and post-treatment glycaemic monitoring among the steroid-treated patients cohort of 29 patients. Baseline fasting glucose before steroid initiation was documented in only 12 of 29 patients (41.4%), and follow-up fasting glucose after steroid completion was documented in even fewer patients, five of 29 (17.2%), as shown in Figure 4. Baseline HbA1c was recorded in 20 patients (69.0%), and follow-up HbA1c was recorded in 18 patients only (62.1%), as shown in Figure 5. 

## Discussion

This retrospective study identified incomplete metabolic monitoring in patients with TED treated with systemic glucocorticoids. Fewer than half of the patients had documented baseline fasting glucose levels, and follow-up fasting glucose levels after steroid completion were rarely recorded. Although HbA1c levels were more frequently documented, they remained incomplete. This finding is clinically significant because glucocorticoid-induced dysglycaemia is common and frequently under-recognized. Multiple practical and societal guidelines recommend baseline glycaemic assessment, including HbA1c where appropriate, and proactive glucose monitoring for patients receiving high-dose systemic steroids [1,3,4,13]. As systemic glucocorticoids remain the first-line therapy for active moderate-to-severe TED according to the EUGOGO guidelines, integrating safety monitoring into the steroid treatment pathway is a critical implementation issue rather than an optional addition [5].

TED can affect up to 40% of patients with Graves‘ disease [14]. TED can lead to inflammation of the eyes, eye muscles, and surrounding tissues, resulting in dry eyes, bulging of the eyes, and double vision, and can have a significant impact on the quality of life [14]. Clinical trials have shown variable results due to glucocorticoid treatment, with response rates ranging from 80% to approximately 20-41% of patients showing no response or relapse of the condition [14]. In terms of severity, 77%, 22%, and 1% of patients had severe, moderate-to-severe, and life-threatening forms of TED, respectively. Furthermore, most patients with TED have Graves' disease; however, approximately 5% of patients may be euthyroid or hypothyroid [15]. Several risk factors have been identified, including older age, male sex, smoking, diabetes mellitus, duration of hyperthyroidism, hypercholesterolaemia, radioactive iodine therapy, and the presence of thyrotropin receptor (TSHR) antibodies (detectable in >95% of patients and directly related to TED activity and severity) [15].

Compared with other centres, the completeness of metabolic monitoring in this cohort appears to be lower than that achievable when surveillance is integrated into treatment workflows. For example, a UK thyroid eye clinic reported protocolized monitoring, with glucose levels measured before treatment and after each dose of intravenous methylprednisolone [16]. Similarly, a tertiary centre Korean Graves’ orbitopathy cohort receiving intravenous methylprednisolone had regular blood glucose monitoring during treatment [17]. Beyond TED, a large multicentre cohort of patients with ophthalmic inflammatory eye disease found that systemic corticosteroids were associated with an increased risk of hyperglycaemia requiring therapy, underscoring the need for structured monitoring in steroid-treated populations with ophthalmic inflammatory eye disease [18]. Additional ophthalmic data on intravenous pulse methylprednisolone further demonstrate clinically meaningful changes in glucose tolerance, supporting the rationale for routine surveillance, particularly among patients with baseline risk factors [19].

Several local service-level factors likely contribute to the observed gaps, such as a lack of standardised local protocols for monitoring glucose and HbA1c in these patients, as well as staff shortages and fragmented responsibility between specialties. These issues are consistent with broader challenges in UK TED services and align with the recommendations for multidisciplinary models described by the British Oculoplastic Surgery Society (BOPSS)/Thyroid Eye Disease Amsterdam Declaration Implementation Group UK (TEAMeD) based on real-world service evaluation data [2,11]. The argument for a systematic metabolic assessment is further supported by emerging TED-specific evidence indicating that HbA1c is associated with clinically important outcomes, such as DON [12]. This would improve patient monitoring, resulting in better outcomes for patients and reducing financial costs for organisations in the long run.

Limitations

This study has several limitations, including being a single-center retrospective study and a lack of a standardised pathway, which limits its generalisability. Furthermore, there is also a risk of information bias in retrospective studies due to reliance on documentation-based ascertainment, and patients may have these tests performed elsewhere, not available on our electronic health records. Follow-up testing was not based on a standard protocol; therefore, the follow-up glucose and HbA1c results obtained were the first measurement available after the initial TED clinic visit. This was not based on a standard protocol, leading to significant variations in patient care. Confounding by indication is also relevant, as steroid-treated patients are likely to have more active or severe diseases. Consequently, these limitations indicate that the findings should be interpreted as describing monitoring completeness rather than quantifying the incidence of steroid-induced hyperglycaemia or related harms.

## Conclusions

Glucose and HbA1c monitoring during systemic glucocorticoid therapy in patients with TED at our centre was significantly below the recommended range, based on national and international guidelines. This was despite the recognised risk of steroid-induced hyperglycaemia and new-onset diabetes with high-dose steroid exposure in patients on high-dose steroids for a longer duration. To improve patient safety and standardise care, we recommend establishing a joint endocrine-ophthalmology “one-stop” TED clinic with a local protocol that mandates baseline and post-treatment fasting glucose and HbA1c assessments for all steroid-treated patients. This would improve patient care through clear responsibility and streamlining of these services by aligning local practice with national guidelines or recommendations. Finally, it can improve patient care by requesting follow-up blood tests, such as HbA1c and glucose levels, for patients attending the TED clinic and making onwards referrals for patients diagnosed with diabetes to the endocrine and diabetes clinics.
